# Effect of preoperative radiotherapy on the prognosis of patients with stage cTxN0M0 esophageal squamous cell carcinoma: propensity score matching analysis based on SEER database

**DOI:** 10.3389/fsurg.2023.1052932

**Published:** 2023-03-21

**Authors:** Zixian Jin, Jiajing Sun, Jian Zhang, Jianfei Shen, Bo Zhang

**Affiliations:** ^1^ Department of Cardiothoracic Surgery, Taizhou Hospital of Zhejiang Province Affiliated to Wenzhou Medical University, Linhai, China; ^2^ Department of Cardiothoracic Surgery, Taizhou Hospital of Zhejiang Province, Zhejiang University, Linhai, China

**Keywords:** ESCC, cN0, PSM, OS, radiotherapy

## Abstract

**Objective:**

The aim of this study was to investigate the effect of preoperative radiotherapy (RT) on overall survival (OS) in patients with stage cTxN0M0 esophageal squamous cell carcinoma (ESCC).

**Methods:**

A total of 467 patients with ESCC diagnosed as cTxN0M0 and undergoing esophagectomy between 2004 and 2016 were downloaded from the Surveillance, Epidemiology, and End Results (SEER) database. According to the presence or absence of preoperative RT, the patients were divided into preoperative RT group and non-preoperative RT group. Propensity score matching (PSM) was performed to equalize baseline levels between groups. Univariate and multivariate Cox regression analyses were used to compare the survival differences between the two groups.

**Results:**

Using PSM, 162 pairs of patients were selected. Preoperative RT was not a prognostic factor for OS in all patients with cTx stage. After PSM, for patients with cT1–2 stage, univariate Cox regression analysis showed that preoperative RT was an influencing factor of OS, and multivariate Cox regression analysis confirmed that preoperative RT was an independent predictor of OS. Compared with non-preoperative RT, preoperative RT significantly decreased OS (HR = 1.556, 95%CI 1.008–2.464, *p* = 0.046). For patients with cT3–4, univariate Cox regression analysis showed that preoperative RT was an influencing factor for OS, and multivariate Cox regression analysis determined that preoperative RT was independent predictors of survival. Compared with non-preoperative RT, preoperative RT significantly improved the OS (HR = 0.479, 95%CI 0.272–0.841, *p* = 0.010).

**Conclusion:**

For ESCC, preoperative RT can improve the OS of patients with cT3-4N0M0. However, preoperative RT is not suitable for patients with cT1-2N0M0.

## Introduction

The incidence of esophageal cancer is increasing year by year (1). At present, the radical treatment of esophageal cancer mainly adopts surgical resection, radiotherapy and chemotherapy. Clinical studies have shown that single treatment can not achieve the ideal treatment effect (2, 3). How to take the comprehensive treatment plan for esophageal cancer is the focus of clinical researchers.

Preoperative neoadjuvant therapy is associated with tumor downstaging and can improve the resection rate and long-term survival rate of esophageal cancer, so it is usually used in patients with locally advanced stage. Several clinical trials have demonstrated the efficacy of preoperative neoadjuvant therapy (4–7). However, given the frequent presence of locally advanced disease and frequent lymph node metastases in these clinical trials, it is difficult to conclude that patients with early stage or non-metastatic lymph nodes would benefit from preoperative neoadjuvant therapy. For these patients, the efficacy of preoperative neoadjuvant therapy remains to be verified.

This study aimed to explore the effect of preoperative radiotherapy (RT) on the prognosis of patients with cTxN0M0 esophageal squamous cell carcinoma (ESCC) through a propensity score matching (PSM) study based on the Surveillance, Epidemiology, and End Results (SEER) database.

## Materials and methods

### Study population

The SEER database is a publicly available database that includes data from 18 cancer registries in the United States, representing approximately 29% of the U.S. population (8). Patients diagnosed with ESCC between 2004 and 2016 were downloaded from the SEER database. Inclusion criteria: (1) Patients undergoing preoperative radiotherapy combined with surgery. (2) Patients with definite preoperative cTNM staging. (3) Stage cN0 was diagnosed without distant metastasis. (4) Report follow-up data and survival status. Exclusion criteria: (1) Patients without surgery. (2) Patients without preoperative radiotherapy. (3) Patients receiving postoperative radiotherapy.

### Study variables

The characteristics analyzed in the current study included age at diagnosis (≤65, >65), sex (male, female), race (white, black and other/unkown), tumor site (upper, middle and lower third), tumor cT stage, histologic grade (high, moderate and poor), follow-up time, and survival status. OS was the end point of the study. OS was defined as the time from diagnosis to death from any reason.

### Statistical analysis

The patients were divided into preoperative RT group and non-preoperative RT group according to the presence or absence of preoperative RT. Statistical analysis was performed using SPSS software. The chi-square test or Fisher exact test was used to compare categorical variables. PSM was used to balance baseline levels between groups (age, sex, race, tumor site, histologic grade) with a caliper value of 0.02. Univariate Cox regression analysis was used to screen the influencing factors of OS. Factors with *p* < 0.1 were included in multivariate Cox regression analysis to determine the independent predictors of OS. The hazard ratio (HR) and its 95% confidence intervals (95%CI) were calculated to assess the strength of association between different characteristics and OS. Subgroup analysis was performed by cT stage (cT1–2, cT3–4) to further determine the factors affecting OS. Kaplan-meier method was used to draw survival curves. A two-sided *p*-value of <0.05 was considered statistically significant.

## Results

### Patient characteristics

**Figure 1:**
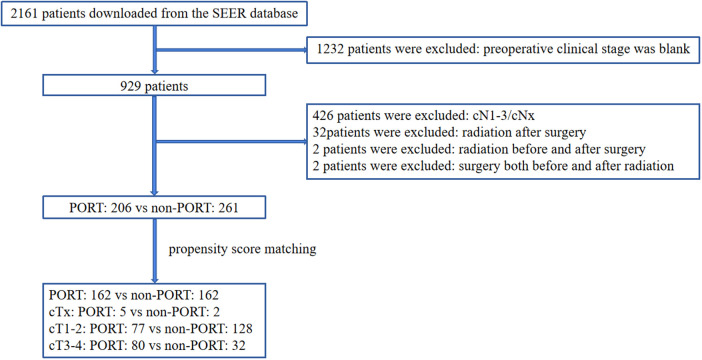
Flow chart of patient inclusion and exclusion.

A total of 2,161 patients diagnosed with ESCC who underwent surgery were downloaded from SEER database. 467 patients were enrolled in this study, including 206 patients who received preoperative RT and 261 patients who did not receive preoperative. The specific process of patient selection is shown in Figure 1. Baseline unadjusted comparisons of patient demographics and oncological outcomes by treatment group (preoperative RT vs. non-preoperative RT) are shown in Table 1. Among the patients with cT3–4, the patients with preoperative RT were more than those without preoperative RT. After PSM, 162 patients were enrolled in each group. The patient demographics and tumor outcomes comparisons between the two groups are shown in Table 2. Similarly, in patients with cT3–4, patients with preoperative RT are more than those without preoperative RT.

**Table 1:** Comparison of patient demographics and tumor characteristics for the clinical node-negative patients before propensity score matching.

Characteristics	preoperative RT (*n* = 206)	Non-preoperative RT(*n* = 261)	*p*
Race, *n* (%)			0.197
White	150	178	
Black	36	43	
Other/unkown	20	40	
Age, years, *n* (%)			0.000
≤65	139	118	
>65	67	143	
Sex, *n* (%)			0.259
Male	115	132	
Female	91	129	
Disease site, *n* (%)			0.919
Upper third	16	24	
Middle third	85	105	
Lower third	79	96	
Other/unkown	26	36	
Histologic grade, *n* (%)			0.099
High	12	32	
Moderate	109	137	
Poor	62	65	
Other/unkown	23	27	
cT stage, *n* (%)			0.000
cT1	54	165	
cT2	42	40	
cT3–4	105	53	
Other/unkown	5	3	
Survival status, *n* (%)			0.557
Alive	112	149	
Dead	94	112	

RT, radiotherapy.

**Table 2:** Comparison of patient demographics and tumor characteristics for the clinical node-negative patients after propensity score matching.

Characteristics	preoperative RT (*n* = 162)	Non-preoperative RT (*n* = 162)	*p*
Race, *n* (%)			0.590
White	114	107	
Black	29	30	
Other/unkown	19	25	
Age, years, *n* (%)			0.494
≤65	96	102	
>65	66	60	
Sex, *n* (%)			1
Male	81	81	
Female	81	81	
Disease site, *n* (%)			0.995
Upper third	13	14	
Middle third	60	59	
Lower third	66	65	
Other/unkown	23	24	
Histologic grade, *n* (%)			0.659
High	11	13	
Moderate	96	85	
Poor	40	45	
Other/unkown	15	19	
cT stage, *n* (%)			0.000
cT1	40	103	
cT2	37	25	
cT3–4	80	42	
Other/unkown	5	2	
Survival status, *n* (%)			0.500
Alive	90	96	
Dead	72	66	

RT, radiotherapy.

## COX regression analysis

### All patients

Preoperative RT was not a significant factor in OS for all patients, regardless of whether PSM was performed for covariates. Table 3 shows the effects of pre-PSM and post-PSM covariates on postoperative OS.

**Table 3:** Univariable cox analysis of the influence of each characteristic on overall survival.

Characteristics	Before PSM		*p*	After PSM		*p*
	HR	95%CI		HR	95%CI	
**Race**						
White	Ref			Ref		
Black	1.281	0.909–1.805	0.157	1.411	0.941–2.116	0.096
Other/unkown	1.240	0.820–1.875	0.307	1.364	0.830–2.243	0.221
**Age, years**						
≤65	Ref			Ref		
>65	1.079	0.821–1.418	0.587	1.041	0.741–1.463	0.817
**Sex**						
Male	Ref			Ref		
Female	0.837	0.636–1.103	0.207	0.978	0.700–1.365	0.894
**Disease site**						
Upper third	Ref			Ref		
Middle third	0.804	0.505–1.280	0.358	0.998	0.535–1.861	0.994
Lower third	0.526	0.323–0.856	0.010	0.733	0.389–1.382	0.337
Other/unkown	0.766	0.440–1.333	0.345	1.120	0.557–2.250	0.751
**Histologic grade**						
High	Ref			Ref		
Moderate	0.491	0.230–1.049	0.066	2.674	1.081–6.616	0.033
Poor	1.296	0.804–2.807	0.287	3.266	1.292–8.254	0.012
Other/unkown	1.560	0.945–2.573	0.082	2.158	0.769–6.057	0.144
**cT stage**						
cT1	Ref			Ref		
cT2	1.127	0.762–1.666	0.549	1.200	0.757–1.903	0.438
cT3–4	1.372	1.012–1.861	0.043	1.380	0.944–2.081	0.096
Other/unkown	1.990	0.729–5.430	0.179	2.369	0.856–6.556	0.097
**Preoperative RT**						
No	Ref			Ref		
Yes	1.111	0.845–1.462	0.450	1.105	0.791–1.543	0.558

RT, radiotherapy; Ref, reference.

### cT1–2

After PSM, 205 patients were in cT1–2 stage, and 77 of them received preoperative RT. Baseline comparisons of patient demographics and oncological outcomes by treatment group are shown in Supplementary Table S1. Baseline characteristics were not significantly unbalanced between the two groups. Univariate Cox regression analysis showed that preoperative RT and histologic grade were the influencing factors of OS, and preoperative RT was associated with increased risk of death (HR = 1.585, 95%CI 1.027–2.446, *p* = 0.037). Multivariate Cox regression analysis also showed that preoperative radiotherapy was an independent risk factor for OS in patients with stage cT1–2 ESCC (HR = 1.556, 95%CI 1.008–2.464, *p* = 0.046). The specific results of Cox analysis are shown in Table 4.

**Table 4:** Univariable and multivariable cox analysis of the influence of each characteristic on overall survival for cT1-2 patients after propensity score matching.

Characteristics	Univariable		*p*	Multivariable		*p*
**Race**						
White	Ref			–		
Black	1.381	0.842–2.264	0.201			
Other/unkown	1.108	0.544–2.257	0.777			
**Age, years**						
≤65	Ref			–		
>65	0.871	0.558–1.360	0.544			
**Sex**						
Male	Ref			–		
Female	1.067	0.692–1.647	0.768			
**Disease site**						
Upper third	Ref			–		
Middle third	0.958	0.398–2.309	0.924			
Lower third	0.962	0.400–2.313	0.931			
Other/unkown	1.679	0.662–4.261	0.276			
**Histologic grade**						
High	Ref			Ref		
Moderate	3.343	1.041–10.732	0.043	3.215	1.001–10.325	0.050
Poor	2.661	0.783–9.041	0.117	2.552	0.751–8.677	0.134
Other/unkown	2.686	0.739–9.765	0.134	2.479	0.680–9.032	0.169
**Preoperative RT**						
No	Ref					
Yes	1.585	1.027–2.446	0.037	1.556	1.008–2.404	0.046

RT, radiotherapy; Ref, reference.

**Figure 2:**
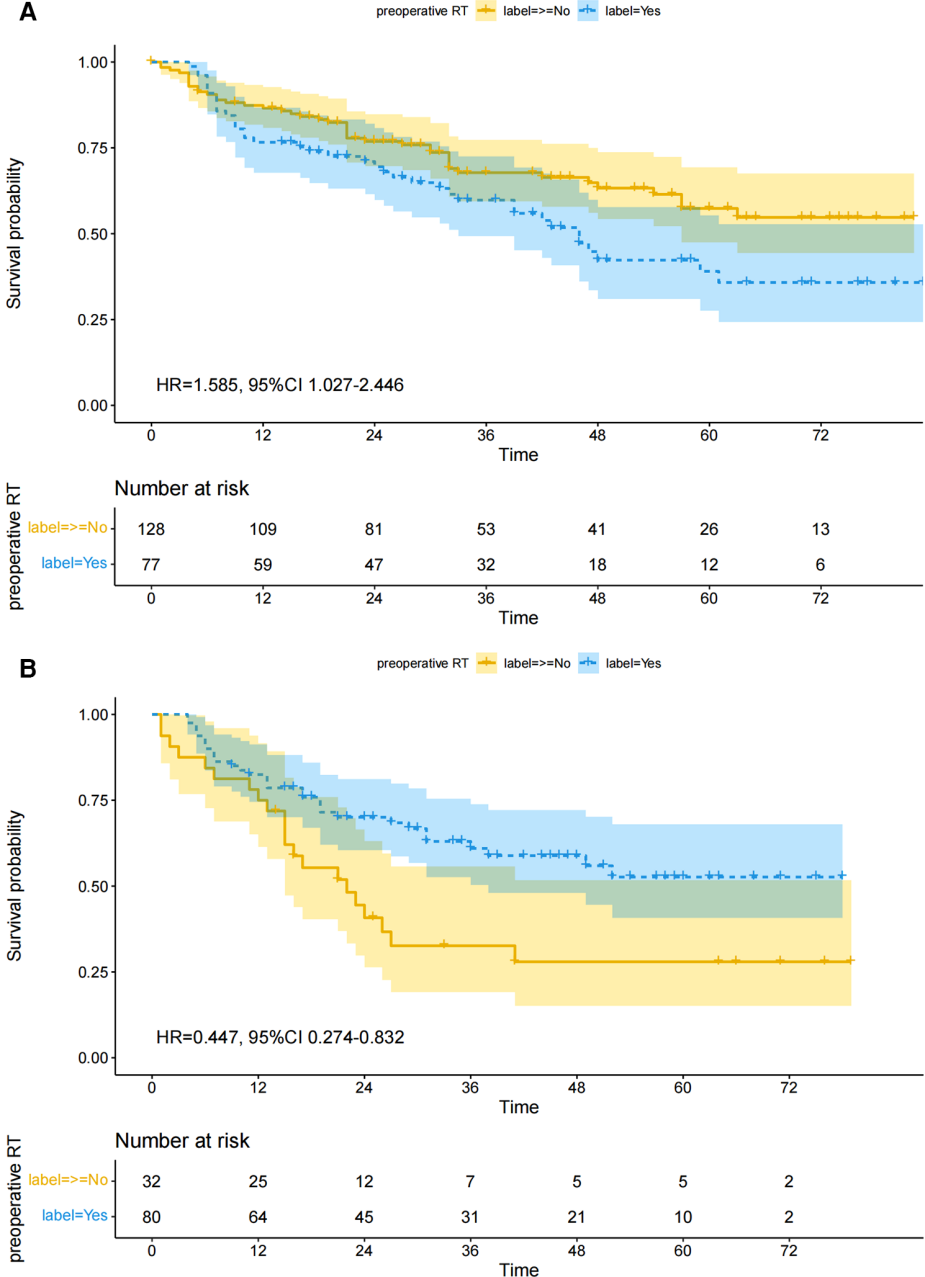
Overall survival between preoperative RT and non-preoperative RT groups after matching. (**A**) cT1–2, (**B**) cT3–4.

The survival curve drawn according to the Kaplan-Meier method is shown in Figure 2A. Preoperative RT increased the overall risk of death in patients with cT1–2. There was no significant difference in 1-year (76.62 vs. 86.72, chi-square = 3.461, *p* = 0.063) and 3-year (62.34 vs. 71.88, chi-square = 2.020, *p* = 0.155) survival between the two groups, but preoperative radiotherapy was associated with a significant reduction in 5-year survival (50.65 vs. 67.19%, chi-square = 5.526, *p* = 0.019).

### cT3–4

After PSM, 112 patients were in cT3–4 stage, and 80 of them received preoperative RT. Baseline comparisons of patient demographics and oncological outcomes by treatment group are shown in Supplementary Table S2. Baseline characteristics were not significantly unbalanced between the two groups. Univariate Cox regression analysis showed that preoperative RT and race were the influencing factors of OS, and preoperative RT was associated with a reduced risk of death (HR = 0.477, 95%CI 0.274–0.832, *p* = 0.009). Multivariate Cox regression analysis also showed that preoperative RT was an independent predictor of OS in patients with cT3–4 ESCC, and preoperative RT significantly reduced the overall risk of death (HR = 0.479, 95%CI 0.272–0.841, *p* = 0.010). The specific results of Cox analysis are shown in Table 5.

**Table 5:** Univariable and multivariable cox analysis of the influence of each characteristic on postoperative survival for cT3-4 patients after propensity score matching.

Characteristics	Univariable		*p*	Multivariable		*p*
**Race**						
White	Ref			Ref		
Black	1.904	0.910–3.981	0.087	1.727	0.823–3.627	0.149
Other/unkown	1.976	0.971–4.018	0.060	2.087	1.020–4.237	0.044
**Age, years**						
≤65	Ref			–		
>65	1.206	0.693–2.099	0.508			
**Sex**						
Male	Ref			–		
Female	0.848	0.489–1.471	0.557			
**Disease site**						
Upper third	Ref			–		
Middle third	1.214	0.464–3.175	0.693			
Lower third	0.574	0.209–1.575	0.281			
Other/unkown	0.810	0.247–2.659	0.729			
**Histologic grade**						
High	Ref			–		
Moderate	1.727	0.408–7.314	0.458			
Poor	3.330	0.786–14.114	0.103			
Other/unkown	0.956	0.134–6.798	0.964			
**Preoperative RT**						
No	Ref			Ref		
Yes	0.477	0.274–0.832	0.009	0.479	0.273–0.841	0.010

RT, radiotherapy; Ref, reference.

The survival curve drawn according to the Kaplan-Meier method is shown in Figure 2B. Preoperative RT reduced the overall risk of death in patients with cT3–4. There was no significant difference in 1-year (82.50 vs. 75.00, chi-square = 0.815, *p* = 0.367) survival between the two groups, but preoperative radiotherapy was significantly associated with improved 3-year (65.00 vs. 37.50, chi-square = 7.058, *p* = 0.008) and 5-year (61.25 vs. 34.38, chi-square = 6.637, *p* = 0.008) survival.

## Discussion

In this propensity score matching study, we found that preoperative RT was associated with OS in patients undergoing surgery for ESCC with preoperative diagnosis of stage cTxN0M0. Especially in patients with cT3–4 stage, preoperative RT produced a significant effect. But for low stage patients (cT1–2), preoperative RT had a negative impact.

Preoperative neoadjuvant therapy has been widely used as a supplement to surgery for esophageal cancer (9, 10). The CROSS trial and the 5010 trial confirmed that preoperative chemoradiotherapy could significantly improve the long-term survival of patients with locally advanced esophageal cancer, and the 5010 trial alone targeted esophageal squamous cell carcinoma (4, 5). A study by the JCOG group in Japan has shown that preoperative chemotherapy also has a good survival effect (6).

For preoperative RT, there were also clinical studies reporting results. For example, the study by Dong et al. showed that preoperative RT improved long-term survival in locally advanced ESCC (11). However, the study of Gao et al. showed that preoperative radiotherapy is only suitable in certain populations (12). This difference means that preoperative radiotherapy is not suitable for all patients. In addition, most of the patients included in the previous clinical trials were locally advanced with lymph node metastasis. Therefore, for patients without lymph node metastasis, the effect of preoperative neoadjuvant therapy is still unclear (13–15). We designed a propensity matching study specifically for patients with ESCC who were preoperatively diagnosed as stage cTxN0M0. Considering that the number of patients diagnosed with stage cN0M0 and receiving preoperative neoadjuvant therapy in a single medical center is small, it is difficult to formulate a valid analysis. Therefore, we downloaded the data of such patients from the SEER database. Due to its population-based nature, there are significant advantages to using the SEER database: the database collects data from 18 registries in 14 U.S. states, representing nearly 30% of the U.S. population, equivalent to a large multicenter database. In addition, treatment decisions for esophageal cancer must be made according to stage, so we also stratified patients according to cT stage to further study the efficacy of preoperative radiotherapy. Theoretically, preoperative treatment can help to shrink the tumor and shrink the lymph node, thereby increasing the radical resection rate and improving the long-term survival rate. However, in practice, comprehensive treatment is very complicated. Preoperative radiotherapy is associated with additional treatment-related adverse effects compared with surgery alone, adversely affecting quality of life in some patients, and potentially increasing postoperative mortality. Our study showed that preoperative RT was not appropriate for all patients with cTxN0M0. Preoperative RT was suitable for patients with stage cT3–4 ESCC, while patients with stage cT1–2 could not benefit from preoperative RT, and preoperative RT had a negative effect on patients with low cT stage. It's not hard to understand. For patients with cT1–2, the probability of occult lymph node metastasis and the depth of tumor invasion is low, and R0 resection is easy to be achieved by surgery. As a result, preoperative radiotherapy cannot bring significant survival effect, and the patients bear potential radiotherapy related adverse reactions. A meta-analysis showed that preoperative neoadjuvant therapy could reduce the tumor stage of cT2N0 stage esophageal cancer, but did not improve patient survival (16). Two multi-center retrospective studies in Taiwan and Europe showed that neoadjuvant therapy provided significant survival benefits for cT3N0 esophageal cancer (17, 18). Similarly, a large retrospective study by Gao et al. showed that although neoadjuvant therapy helped to improve postoperative survival in esophageal cancer patients with cN0 on the whole, neoadjuvant therapy was associated with decreased survival for early-staged true node-negative patients (12).

Of course, there are some limitations in this study. First, our results were based on a retrospective study. We grouped patients according to treatment mode and were therefore not random, which could lead to selection bias. Second, although propensity matching was used to avoid the imbalance between groups as much as possible, due to the limitations of the database itself, other data that might affect survival (such as comorbidities, physical status, etc.) were not available. Moreover, we do not have the exact treatment data of the patients, such as the specific dose and regimen of radiotherapy. Radiotherapy regimens and methods have been rapidly developed in the past decades, such as 3-dimensional conformal radiation therapy (3D-CRT) and intensity modulated radiation therapy (IMRT), and their efficacy has been proven (19–21). In addition, due to limited access to the database, it was not possible to know whether patients receiving preoperative radiation therapy included some patients receiving salvage surgery after radiotherapy. Patient survival depends on the treatment techniques and regimens used, and further research is needed in this aspect. And, due to the limitation of the number of people diagnosed with cN0 stage in the SEER database from 2004 to 2016, the number of patients included in this article was not large. As the database data is constantly updated, we will dig deeper.

## Conclusion

For ESCC, preoperative RT can improve the OS of patients with cT3-4N0M0, which is worthy of clinical application. However, preoperative RT is not suitable for patients with cT1-2N0M0. The role of preoperative RT should be further investigated in prospective studies.

## Data Availability

The original contributions presented in the study are included in the article/**Supplementary Material**, further inquiries can be directed to the corresponding author/s.
