# Supplementary material for: Effect of preoperative radiotherapy on the prognosis of patients with stage cTxN0M0 esophageal squamous cell carcinoma: propensity score matching analysis based on SEER database
Source: Front Surg. 2023 Mar 21;10:1052932. doi: 10.3389/fsurg.2023.1052932 (PMC10070869; doi:10.3389/fsurg.2023.1052932)
Supplement: Supplementary file 2 [file Table2.docx]

**Supplementary Table 1.** Baseline comparison of demographics and oncological outcomes in cT1-2 patients.

| Characteristics | PRRT(n=77) | Non-PRRT(n=128) | p |
| --- | --- | --- | --- |
| Race, n(%) |  |  | 0.164 |
| White | 50 | 83 |  |
| Black | 21 | 25 |  |
| Other/unkown | 6 | 20 |  |
| Age, years, n (%) |  |  | 0.292 |
| ≤65 | 43 | 81 |  |
| ＞65 | 34 | 47 |  |
| Sex, n (%) |  |  | 0.241 |
| Male | 32 | 64 |  |
| Female | 45 | 64 |  |
| Disease site, n (%) |  |  | 0.736 |
| Upper third | 5 | 11 |  |
| Middle third | 31 | 45 |  |
| Lower third | 28 | 54 |  |
| Other/unkown | 13 | 18 |  |
| Histologic grade, n (%) |  |  | 0.976 |
| High | 6 | 10 |  |
| moderate | 45 | 73 |  |
| Poor | 18 | 29 |  |
| Other/unkown | 8 | 16 |  |
| Survival status, n (%) |  |  |  |
| Alive | 38 | 85 | 0.016 |
| Dead | 39 | 43 |  |

PRRT: preoperative radiotherapy.

**Supplementary Table 2.** Baseline comparison of demographics and oncological outcomes in cT3-4 patients.

| Characteristics | PRRT(n=80) | Non-PRRT(n=32) | p |
| --- | --- | --- | --- |
| Race, n(%) |  |  | 0.714 |
| White | 59 | 22 |  |
| Black | 8 | 5 |  |
| Other/unkown | 13 | 5 |  |
| Age, years, n (%) |  |  | 0.666 |
| ≤65 | 49 | 21 |  |
| ＞65 | 31 | 11 |  |
| Sex, n (%) |  |  | 0.471 |
| Male | 46 | 16 |  |
| Female | 34 | 16 |  |
| Disease site, n (%) |  |  | 0.424 |
| Upper third | 7 | 3 |  |
| Middle third | 28 | 13 |  |
| Lower third | 37 | 10 |  |
| Other/unkown | 8 | 6 |  |
| Histologic grade, n (%) |  |  | 0.188 |
| High | 5 | 3 |  |
| moderate | 46 | 12 |  |
| Poor | 22 | 15 |  |
| Other/unkown | 7 | 2 |  |
| Survival status, n (%) |  |  | 0.010 |
| Alive | 49 | 11 |  |
| Dead | 31 | 21 |  |

PRRT: preoperative radiotherapy.
